# Parasites and blood-meal hosts of the tsetse fly in Tanzania: a metagenomics study

**DOI:** 10.1186/s13071-022-05344-1

**Published:** 2022-06-22

**Authors:** Ju Yeong Kim, Jun Ho Choi, Sung-Hyun Nam, Robert Fyumagwa, Tai-Soon Yong

**Affiliations:** 1 Department of Environmental Medical Biology, Institute of Tropical Medicine and Arthropods of Medical Importance Resource Bank, Yonsei University College of Medicine, Seoul, 03722 Republic of Korea; 2 Brain Korea 21 Plus Project for Medical Sciences, Yonsei University College of Medicine, Seoul, 03722 Republic of Korea; 3 Tanzania Wildlife Research Institute, P.O. Box 661, Arusha, Tanzania

**Keywords:** Amplicon deep sequencing, *Trypanosoma*, Trypanosomiasis, Tsetse fly, Tanzania

## Abstract

**Background:**

Tsetse flies can transmit various *Trypanosoma* spp. that cause trypanosomiasis in humans, wild animals, and domestic animals. Amplicon deep sequencing of the 12S ribosomal RNA (rRNA) gene can be used to detect mammalian tsetse hosts, and the 18S rRNA gene can be used to detect all associated eukaryotic pathogens, including *Trypanosoma* spp.

**Methods:**

Tsetse flies were collected from the Serengeti National Park (*n* = 48), Maswa Game Reserve (*n* = 42), and Tarangire National Park (*n* = 49) in Tanzania in 2012–13. Amplicon deep sequencing targeting mammal-specific 12S rRNA and 18S rRNA genes was performed to screen the blood-feeding sources of tsetse flies and eukaryotic parasites in tsetse flies, respectively.

**Results:**

12S rRNA gene deep sequencing revealed that various mammals were blood-feeding sources of the tsetse flies, including humans, common warthogs, African buffalos, mice, giraffes, African elephants, waterbucks, and lions. Genes of humans were less frequently detected in Serengeti (*P* = 0.0024), whereas African buffaloes were detected more frequently as a blood-feeding source (*P* = 0.0010). 18S rRNA gene deep sequencing showed that six tsetse samples harbored the *Trypanosoma* gene, which was identified as *Trypanosoma godfreyi* and *Trypanosoma simiae* in subsequent ITS1 gene sequencing.

**Conclusions:**

Through amplicon deep sequencing targeting the 12S rRNA and 18S rRNA genes, various mammalian animals were identified as blood-meal sources, and two *Trypanosoma* species were detected in tsetse flies collected from the Maswa Game Reserve, Serengeti National Park, and Tarangire National Park in Tanzania. This study illustrates the patterns of parasitism of tsetse fly, wild animals targeted by the fly, and *Trypanosoma* spp. carried by the fly in Tanzania. It may provide essential data for formulating better strategies to control African trypanosomes.

**Graphical Abstract:**

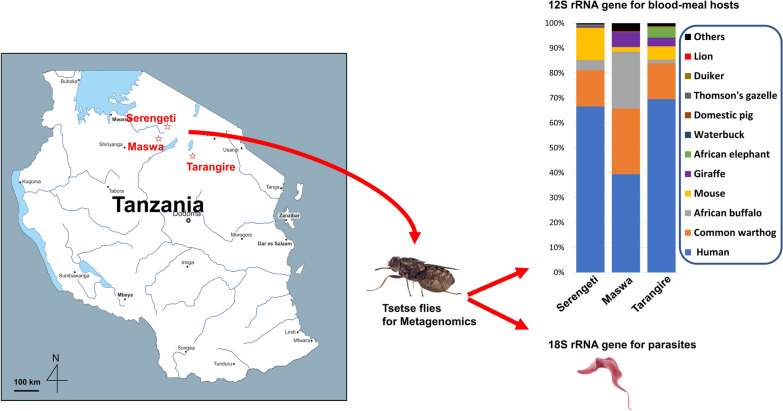

**Supplementary Information:**

The online version contains supplementary material available at 10.1186/s13071-022-05344-1.

## Background

The tsetse fly is a vector of *Trypanosoma brucei gambiense* and *T.b. rhodesiense*, both of which can cause African human trypanosomiasis (HAT) (also known as sleeping sickness). The tsetse fly is also a vector of *T. vivax*, *T. simiae*, *T.b. brucei*, and *T. congolense*, all of which can cause trypanosomiasis in wild and domestic animals (AAT) [1]. HAT and AAT are severe and sometimes fatal diseases affecting the central nervous system in humans and the nervous system and muscles in animals, respectively. The spread of AAT consequently puts a significant constraint on the development of animal husbandry in Africa while HAT is a severe public health problem. As adult tsetse flies are frequently found on cattle and horses, among other animals, and feed on animal blood, they can carry parasites that cause HAT and AAT. There is no cure for either disease, and the only method of prevention involves controlling tsetse flies [2, 3].

Preference of tsetse flies for blood-feeding hosts can vary significantly depending on the species, wildlife, and geographic location [4, 5]. Tsetse flies feed on various wild and domestic mammals [6]. For example, humans, cattle, dogs, bush pigs, African buffaloes, warthogs, greater kudus, rats, and bats were confirmed as the blood-meal sources of tsetse flies in Zambia, using 12S ribosomal RNA (rRNA) gene deep sequencing [7]. Analysis of tsetse flies using vertebrate cytochrome c oxidase I (COX1) and cytochrome b gene polymerase chain reaction (PCR) revealed that humans are the most common vertebrate hosts [8]. This also indicated that other wild species, such as hippopotamuses, African buffaloes, African savannah elephants, and giraffes, may be involved in trypanosomiasis transmission. Blood meal collection and identification are essential for determining the hosts of tsetse flies for epidemiological studies and controlling their population. A study, in which the blood of vertebrates was analyzed in tsetse flies, revealed that the changes in the environment, fauna, and host availability can affect tsetse feeding patterns [9].

The internal transcribed spacer (ITS) region of ribosomal DNA (rDNA) is commonly used to detect *Trypanosoma* spp. in tsetse flies because of the highly conserved flanking regions and size variability between *Trypanosoma* spp. and their subgroups [10–12]. Recently, attempts have been made to detect *Trypanosoma* spp. by using the 18S rRNA region [6, 13]. The 18S rRNA region in eukaryotes is highly conserved across species and allows the detection of a variety of eukaryotic organisms [14–16].

The recent development of high-throughput sequencing allows the use of a metagenomic approach to detect all prokaryotic and eukaryotic species in a sample in a single sequencing run at a low cost [17–19]. In this study, we used amplicon deep sequencing of the 12S rRNA and 18S rRNA genes to identify the mammalian hosts of the tsetse fly and associated eukaryotic pathogens (including *Trypanosoma* spp.), respectively. This efficient, inexpensive, and sensitive method for monitoring biodiversity may provide essential information for formulating new strategies to control tsetse flies in Africa.

## Methods

### Sample collection and identification of tsetse flies

We collected tsetse flies between January 2012 and February 2013 from the Serengeti National Park (*n *= 48), Maswa Game Reserve (*n* = 42), and Tarangire National Park (*n* = 49) in Tanzania. The samples were collected within a 5-km radius of our accommodation (Serengeti National Park-2.434974741607467, 34.85272334722886; Maswa Game Reserve-3.2568282434634996, 34.595773504540574; Tarangire National Park-3.991658476578967, 35.96541568649041). The tsetse flies were caught using a net mounted on the back of a moving vehicle and preserved in absolute ethanol. DNA was extracted from each tsetse fly using a Nucleospin Tissue Kit (Macherey-Nagel, Düren, Germany) according to the manufacturer’s instructions and stored in a deep freezer until testing. Molecular identification of tsetse flies was performed by ITS2 gene amplification and sequencing [20].

### Illumina sequencing and bioinformatics

The 18S rRNA V9 region was identified as 1391f (5'-TCGTCGGCAGCGTCAGATG TGTATAAGAGACAG GTACACACCGCCCGTC-3') and EukBr (5'-GTCTCGTGGG CTCGGAGATGTGTATAAGAGACAGTGATCCTTCTGCAGGTTCACCTAC-3') [17]. The 12S rRNA genes were identified as L1085 (5'-TCGTCGGCAGCGTCAGATGTGTATAAGAGACAGCCCAAACTGGGATTAGATAACCC-3') and H1259 (5'-GTCTCGTGGGCTCGGAGATGTGTATAAGAGACAGGTTTGCTGAAGATGGCGGTA-3') [18]. A limited-cycle (eight cycles) amplification step was performed to add multiplexing indices and Illumina sequencing adapters. Mixed amplicons were pooled and sequenced on an Illumina iSeq 100 sequencing system using the Illumina iSeq™ 100 i1 Reagent v2 kit (San Diego, CA, USA) according to the manufacturer’s instructions.

Geneious Prime^®^ 2022.0.2 (Biomatters Ltd., Auckland, New Zealand) was used to process and assemble raw 18S V9 and 12S rRNA reads in the following steps [21, 22]. Sequences < 100 bp were deleted, and 151 bp regions were amplified. The forward and reverse reads were merged to produce a single consensus sequence. Closely related sequences were clustered into separate contigs using de novo assembly. We used the default setting according to the online manual (https://www.geneious.com/tutorials/metagenomic-analysis/), using ‘Minimum Overlap Identity’ as 98%. Operational taxonomic units (OTUs) were defined via sequence clustering using Basic Local Alignment Search Tool (BLAST) on NCBI “nt” GenBank database (November 2021 version). A curated database was created for taxonomic classification. BLAST hits were used to create a sequence classification database. Lastly, the extracted BLAST hits were assigned the name of the source organism.

### Polymerase chain reaction and sequencing analysis

PCR was performed to identify *Trypanosoma* spp. using the following primer sets: ITS1 CF (5'-CCGGAAGTTCACCGATATTG-3') and ITS1 BR (5'-TTGCTGCGTTCTTCAACGAA-3') [10]. Sequencing of positive PCR amplicons was performed by Bionics Co., Ltd. (Seoul, Korea). A BLAST search was used.

To compare the sequence of the obtained ITS1 gene with the series available in GenBank, the obtained sequence was compared to the line deposited in GenBank using BLAST. Gene sequences, except for the primer regions, were aligned using the Multisequence Alignment Program (Geneious).

## Results

Among the 139 tsetse flies collected from the Maswa Game Reserve (*n* = 48), Serengeti National Park (*n* = 42), and Tarangire National Park (*n *= 49), 2 tsetse flies in Tarangire National Park (T51 and T52) were identified as *Glossina morsitans*, and the remaining samples were identified as *Glossina swynnertoni.*

**Fig. 1:**
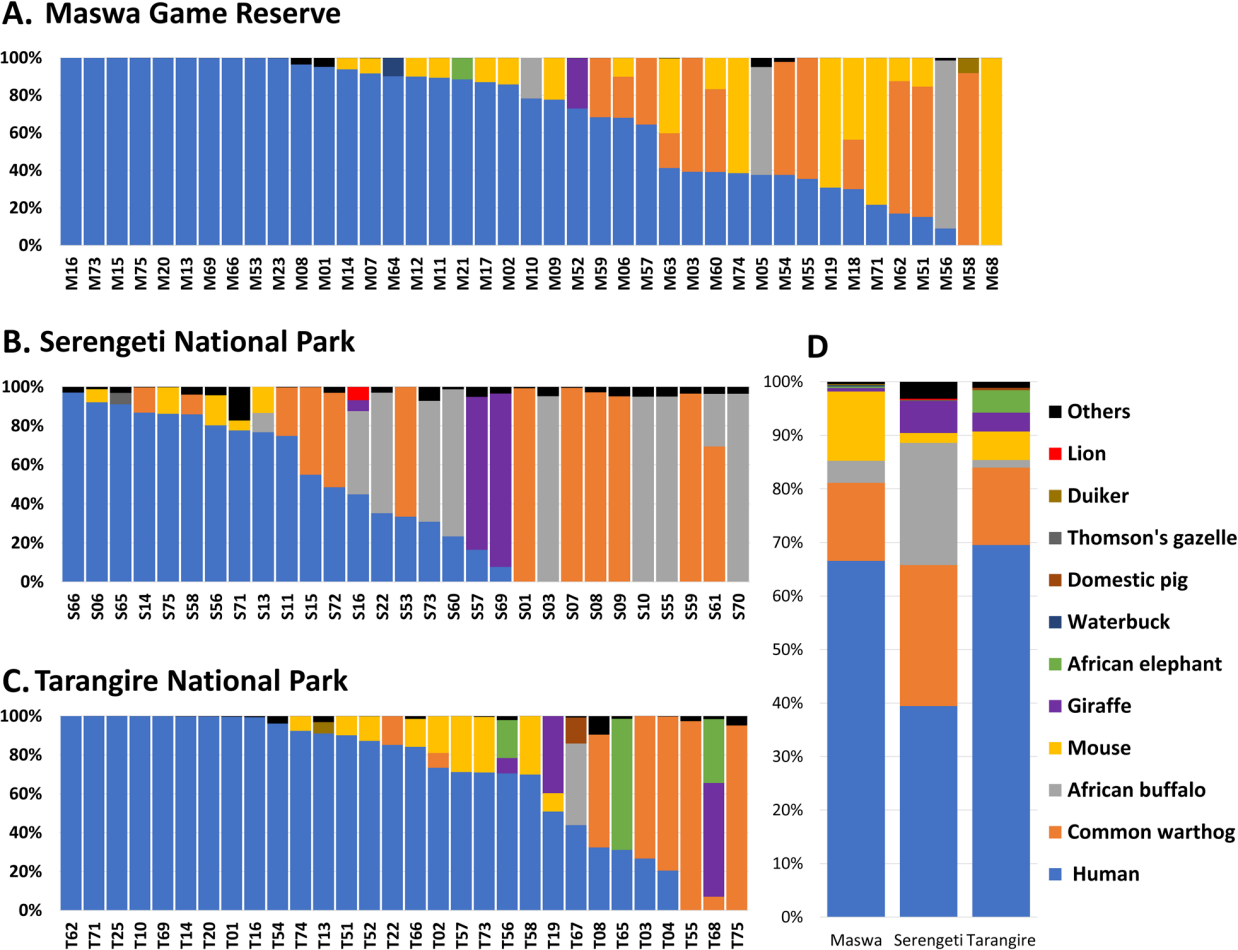
Composition of tsetse fly blood-meal sources collected in the **A** Maswa Game Reserve (*N* = 41), **B** Serengeti National Park (*N* = 29), and **C** Tarangire National Park (*N* = 30). Mammalian-specific 12S rRNA gene deep sequencing was performed for each fly sample. **D** The average relative abundance of tsetse fly blood-meal sources in the Maswa Game Reserve, Serengeti National Park, and Tarangire National Park. Taxa with < 5% relative abundance are included in ‘Others’

Amplicon deep sequencing targeting a mammalian-specific 12S rRNA gene was performed to determine the blood-meal sources of collected tsetse flies. Only 100 samples (41 from Maswa, 29 from Serengeti, and 30 from Tarangire) successfully underwent sequencing and bioinformatics analysis. Various mammalian genes were detected, including those of humans, common warthogs, African buffaloes, mice, giraffes, African elephants, waterbucks, domestic pigs, Thomson’s gazelles, duikers, and lions (Fig. 1). Human genes were primarily found in the tsetse flies of all three regions: 39 (95.12%) in Maswa, 19 (65.52%) in Serengeti, and 27 (90.00%) in Tarangire (Table 1). Genes of humans, common warthogs, African buffaloes, mice, and giraffes were detected in all three regions. In particular, fewer human genes were detected in Serengeti (*P* = 0.0024), while the African buffalo was identified as a blood-meal source here (*P* = 0.0010). In the Maswa Game Reserve, genes of one African elephant, one waterbuck, and one duiker were detected. Genes of one Thomson’s gazelle and one lion were detected in Serengeti National Park. Genes of four African elephants and one domestic pig were detected in Tarangire National Park. There were many samples in which the genes of several mammals were simultaneously detected, particularly human genes (Fig. 1; Table 1). In addition, differences in animal blood-meal sources between male and female tsetse flies were not observed (Additional file 1).

**Table 1:** Number of tsetse flies harboring each animal’s 12S ribosomal RNA genes in the Maswa Game Reserve, Serengeti National Park, and Tarangire National Park

Species	Maswa Game Reserve *N* (%)	Serengeti National Park *N* (%)	Tarangire National Park *N* (%)	*P-*value
Human	39 (95.12%)	19 (65.52%)	27 (90%)	0.0024
Common warthog	12 (29.27%)	12 (41.38%)	8 (26.67%)	0.4516
African buffalo	3 (7.32%)	10 (34.48%)	1 (3.33%)	0.0010
Mouse	17 (41.46%)	5 (17.24%)	9 (30%)	0.0997
Giraffe	1 (2.44%)	3 (10.34%)	3 (10%)	0.3268
African elephant	1 (2.44%)	0 (0%)	4 (13.33%)	0.0613
Waterbuck	1 (2.44%)	0 (0%)	0 (0%)	1
Domestic pig	0 (0%)	0 (0%)	1 (3.33%)	0.5900
Thomson’s gazelle	0 (0%)	1 (3.45%)	0 (0%)	0.2900
Duiker	1 (2.44%)	0 (0%)	0 (0%)	1
Lion	0 (0%)	1 (3.45%)	0 (0%)	0.2900

The total number of screened tsetse flies was 41 in the Maswa Game Reserve, 29 in the Serengeti National Park, and 30 in the Tarangire National Park. Fisher’s exact test was performed. Statistical significance was set at *P* < 0.05.

**Fig. 2:**
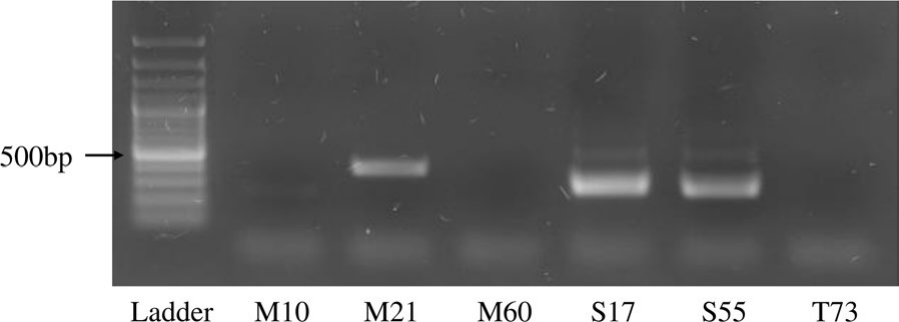
*Trypanosoma*-specific PCR targeting the ITS1 region. The M10, M21, and M60 samples were tsetse flies collected from the Maswa Game Reserve; S17 and S55 were from Serengeti National Park; T73 was from Tarangire National Park

Amplicon sequencing targeting the 18S rRNA V9 region was performed to screen for eukaryotic pathogens, including *Trypanosoma* spp., and 139 samples were successfully sequenced and analyzed. Of these, six tsetse samples harbored *Trypanosoma* genes: three from Maswa, two from Serengeti, and one from Tarangire (Table 2). In addition, human, fungal, and plant genes were detected (Table 2). *Trypanosoma*-specific PCR targeting the ITS1 region, conventional DNA sequencing, and homology analysis were performed for six samples to determine the species. Two samples from Serengeti were identified as *Trypanosoma godfreyi* and one sample from Maswa was identified as *Trypanosoma simiae* (Fig. 2; Table 3)*.* The remaining three samples were not analyzed.

**Table 2:** Read counts of eukaryotic organisms in tsetse flies harboring *Trypanosoma*, which were analyzed through amplicon deep sequencing of the 18S ribosomal RNA gene V9 region

No	Sample ID	*Glossina* spp.	*Trypanosoma* spp.	Human	Fungi	Plant	Total
1	M10	33585	9	2	1	2	33615
2	M21	32750	228	0	1	0	32996
3	M60	27742	1	0	0	0	27758
4	S17	17392	81	0	0	0	17475
5	S55	8736	11	0	0	0	8747
6	T73	34365	2	0	1	0	34389

The identified fungal taxa of M10 and M21 were *Malassezia* and that of T73 was *Arthrocatena.*

**Table 3:** Molecular identification of *Trypanosoma* species with the ITS1 gene

No.	Sample ID	Identification of *Trypanosoma* sp. (accession, similarity %)	Host	Region	Sex of the tsetse fly
1	M10	–*	Human African buffalo	Maswa Game Reserve	Female
2	M21	*Trypanosoma simiae* (JN673382, 100%)	Human African elephant	Maswa Game Reserve	Female
3	M60	PCR failed	Human Common warthog Mouse	Maswa Game Reserve	Male
4	S17	*Trypanosoma godfreyi* (JN673383, 95.3%)	NGS failed	Serengeti National Park	Female
5	S55	*Trypanosoma godfreyi* (MK131836, 99.4%)	African buffalo	Serengeti National Park	Male
6	T73	PCR failed	Human Mouse	Tarangire National Park	Male

PCR was performed with primers targeting the *Trypanosoma*-specific ITS1 gene, and conventional DNA sequencing was performed to identify *Trypanosoma* species

*DNA sequencing failed because of weak amplicon band

## Discussion

Of the 139 tsetse flies collected in Tanzania, 2 were identified as *Glossina morsitans*, and the remaining samples were identified as *Glossina swynnertoni*. This is similar to a previous study analyzing 21,107 tsetse flies which reported that the major tsetse fly species was *G. swynnertoni* (55.9%), while *G. morsitans* (6.0%) was less prevalent [23].

Amplicon deep sequencing was performed using the mammalian-specific 12S rRNA gene to determine the sources of tsetse blood meals. PCR sequencing has previously been performed to determine the species of tsetse flies [24]; however, analyzing the genes of all the collected samples by sequencing requires considerable time and effort. Therefore, deep amplicon sequencing has more recently been utilized to analyze the 12S rRNA gene region of vertebrates [7, 18, 25–27].

Analysis of humans, cattle, dogs, bush pigs, African buffaloes, warthogs, greater kudus, rats, and bats was necessary to confirm the sources of tsetse fly blood meals using 12S rRNA gene deep sequencing [7]. Various mammalian genes including those of humans, common warthogs, African buffaloes, mice, giraffes, African elephants, waterbucks, domestic pigs, Thomson's gazelles, duikers, and lions were detected in our tsetse fly samples. This is consistent with previous studies that identified blood-meal sources by analyzing the mitochondrial cytochrome b gene and indicated that humans, hippopotamuses, African buffaloes, African savannah elephants, and giraffes are reservoirs for trypanosomiasis transmission [28, 29].

In Serengeti, the African buffalo was found to be a more significant blood-meal source than in Maswa and Tarangire (Table 1). The African buffalo gene was found in 34.48% of tsetse flies in Serengeti and 7.32% and 3.33% of tsetse flies in Maswa and Tarangire, respectively. This shows that the African buffalo is a major blood-meal source for tsetse flies in Serengeti. In contrast, humans were found to be a significantly less common bloodmeal source in Serengeti. Tsetse flies likely have a greater chance of contact with wild animals such as African buffaloes and common warthogs in Serengeti than that in the other two regions.

Human genes were found in all three of the Maswa Game Reserve, Serengeti National Park, and Tarangire National Park. Several mammalian genes are commonly simultaneously detected in individual flies [18, 25]. The diet of tsetse flies may change if there are not enough animals from which they can draw blood or if there are houses nearby [7, 9]. This means that tsetse flies will suck human blood if given the opportunity.

The *Trypanosoma* spp. gene was identified by 18S rRNA gene deep sequencing. PCR targeted ITS1 confirmed that two samples from the Serengeti National Park were identified as *Trypanosoma godfreyi*, and one sample from the Maswa Game Reserve was identified as *Trypanosoma simiae* (Fig. 2; Table 3). *Trypanosoma simiae* usually infects pigs [11], and *T. godfreyi* usually infects cattle. In our study, *T. godfreyi* was detected in the tsetse fly containing the African buffalo gene and two *T. godfreyi* samples were detected in flies from Serengeti, where the major blood-meal source is African buffaloes.

There are no reports of *Trypanosoma* spp. found in this study that infect humans. Trypanosoma infection in animals causes red blood cell phagocytosis and blood catabolism, leading to the accumulation of iron in tissues, hyperbilirubinemia, liver dysfunction, and multiple organ failure [30]. Trypanosomiasis, induced by tsetse blood-feeding, makes animals ill; cattle that are protected from trypanosomiasis are healthier and have significantly reduced disease levels, increased cell volume, and greater body weight [31].

Six samples were positive for *Trypanosoma* using 18S rRNA gene deep sequencing, and three samples (M21, S17, and S55) were found to be positive using PCR and subsequent DNA sequencing analysis. One sample (M10) showed a very weak band after PCR amplification, and the DNA sequencing of this sample failed. Two other samples (M60 and T73) were found to be negative using PCR, and the samples showed only one and two *Trypanosoma* reads in deep sequencing, respectively (Table 2). Samples in which *Trypanosoma* was detected with fewer than ten read counts using deep sequencing were not well detected using PCR. This was probably because deep amplicon sequencing is more sensitive than PCR [32, 33]. Therefore, deep sequencing of the 18S rRNA gene is useful for screening for eukaryotic pathogens in tsetse flies.

In addition, because the primers we used can detect all species of eukaryotic organisms, this method can be applied to screen eukaryotic pathogens in any arthropod vector. Our method can theoretically detect all potentially pathogenic taxa in samples and simultaneously analyze 96 samples at once. The approximate cost of one run of the iSeq 100 machine is US$ 2000, which takes 18 h to complete [19].

As we collected tsetse flies while moving by vehicle between villages, we believe that a large number of tsetse flies that sucked the blood of humans were collected. Because many previous studies reported human as a major blood-meal source of tsetse flies, the possibility of contamination during collection was low [7, 24, 34]. The possibility of degradation of the nucleic acid over a long storage period cannot be ruled out, which might reduce the diversity of animal blood-meal sources of tsetse flies.

## Conclusions

Various mammals were identified as blood-meal sources for tsetse flies through 12S rRNA gene deep sequencing, and two species of *Trypanosoma* spp. that infect animals were identified in tsetse flies through 18S rRNA gene deep sequencing in the Maswa Game Reserve, Serengeti National Park, and Tarangire National Park in Tanzania. This study provides important information on the patterns of parasitism of tsetse flies, affected wild animals, and *Trypanosoma* spp. in this region.

## Supplementary Information

**Additional file 1. **The number of tsetse flies harboring 12S ribosomal RNA genes from several animals.

## Data Availability

Raw sequence data are available in NCBI GenBank under BioProject PRJNA817381.

## Abbreviations

AAT: Trypanosomiasis in animals
HAT: African human trypanosomiasis
ITS: Internal transcribed spacer
OUT: Operational taxonomic unit
PCR: Polymerase chain reaction
rRNA: Ribosomal RNA
