# Supplementary material for: Parasites and blood-meal hosts of the tsetse fly in Tanzania: a metagenomics study
Source: Parasit Vectors. 2022 Jun 22;15:224. doi: 10.1186/s13071-022-05344-1 (PMC9215111; doi:10.1186/s13071-022-05344-1)
Supplement: Supplementary file 1 — Additional file 1. The number of tsetse flies harboring 12S ribosomal RNA genes from several animals. [file 13071_2022_5344_MOESM1_ESM.docx]

Additional file 1. The number of tsetse flies harboring 12S ribosomal RNA genes from several animals.

| Species | Male, N (%) | Female, N (%) | *P*-value |
| --- | --- | --- | --- |
| Human | 44 (83.02%) | 41 (87.23%) | 0.5886 |
| Common warthog | 17 (32.08%) | 15 (31.91%) | 1 |
| African buffalo | 7 (13.21%) | 7 (14.89%) | 1 |
| Mouse | 17 (32.08%) | 14 (29.79%) | 0.8318 |
| Giraffe | 5 (9.43%) | 2 (4.26%) | 0.4424 |
| African elephant | 3 (5.66%) | 2 (4.26%) | 1 |
| Waterbuck | 1 (1.89%) | 0 (0%) | 1 |
| Domestic pig | 1 (1.89%) | 0 (0%) | 1 |
| Thomson's gazelle | 1 (1.89%) | 0 (0%) | 1 |
| Duiker | 1 (1.89%) | 0 (0%) | 1 |
| Lion | 0 (0%) | 1 (2.13%) | 0.47 |

The total number of screened male and female flies were 53 and 47, respectively. The Fisher’s exact test was performed.

ㄴ
